# Publically Misfitting: Extreme Weight and the Everyday Production and Reinforcement of Felt Stigma

**DOI:** 10.1111/maq.12309

**Published:** 2016-08-25

**Authors:** Alexandra Brewis, Sarah Trainer, SeungYong Han, Amber Wutich

**Affiliations:** ^1^ School of Human Evolution and Social Change Arizona State University; ^2^ Obesity Solutions Arizona State University; ^3^ Obesity Solutions Arizona State University; ^4^ School of Human Evolution and Social Change Arizona State University

**Keywords:** obesity, stigma, embodiment, weight, ethnography

## Abstract

Living with extreme weight in the United States is associated with discrimination and self‐stigma, creating structural exclusions, embodied stress, and undermining health and wellbeing. Here we combine ethnographic interviews and surveys from those with experiences of living with extreme weight to better explain how this vulnerability is created and reinforced by public cues, both physical (e.g., seatbelts) and social (the reactions of strangers). “Misfitting” is a major theme in interviews, as is the need to plan and scan constantly while navigating too‐small public spaces. The most distressing events combine physical misfitting with unsympathetic reactions from strangers. Sensitivity to stigmatizing public cues reduces with weight loss, but does not disappear. This study explains one basic mechanism that underlies the creation of felt stigma related to weight even after weight loss: the lack of accommodation for size and the lack of empathy from others that characterize modern urban spaces.

The body is how we come to know the world, as we experience it through our perceptions and senses, and for this reason, social scientists from a variety of backgrounds have long studied both the body and processes of embodiment (most famously through Bourdieu [1984, 1992, 1997] and Foucault [1983, 1986]). Medical anthropologists specifically have concerned themselves with issues surrounding the social production of health and disease, including the ways in which structural factors (such as national policies) can generate different forms of discrimination that restrict and block access to health‐related resources, discourage treatment‐seeking, and—more generally—simply incorporate biologically the social and material worlds in which they live (e.g., Csordas 1993, 1994, 2002; Hruschka et al. 2005; Kleinman 1986, 1992; Krieger 2001, 2005; Lende 2012; Manderson 2011; Scheper‐Hughes and Lock 1987; Tapias 2006). In this article, we focus on the ways a stressful “misfit” between the physical environment and the “fat” body in the United States contributes to the production of felt weight‐related stigma and the suffering that accompanies them.

Stigma may be experienced indirectly, via structurally based institutional discriminations, or directly, via interpersonal interactions such as being ignored or teased (Hatzenbuehler et al. 2013; Link and Phelan 2001, 2006; Martin et al. 2008; Pescosolido 2013). Both can potentially create the types of stress and distress that, over the long term, become embedded and incorporated into the body, producing health differentials (Horton and Barker 2010). “Fat stigma” specifically (also termed “obesity stigma” or “weight‐related stigma” in the relevant literatures) negatively influences health via both of these and other mechanisms (see, e.g., Brewis 2014). In particular, *feeling* stigmatized and being explicitly aware of exclusions or mistreatments because of one's “fatness” predicts greater stress, fewer health‐enhancing behaviors, and worsened health outcomes. In other words, as fat studies scholars and anthropologists working within critical fat/obesity studies have pointed out, many of the health issues attributed to those of large body size stem from discrimination and stress resulting from stigma—not necessarily because larger body size itself is inherently diseased (e.g., Braziel and Lebesco 2001; Lupton 2013; McCullough 2013; Owen 2015; Rothblum and Solovay 2009; Yates‐Doerr 2012, 2015).

Fat stigma in the United States today is acutely felt by those it touches, and its reach is extremely broad, given its entrenchment across all sectors of U.S. society (Braziel and Lebesco 2001; Farrell 2011; Greenhalgh and Carney 2014; McCullough and Hardin 2013; Puhl and Heuer 2009; Rogge 2004; Rothblum and Solovay 2009; Tomiyama et al. 2015; Trainer et al. 2015a, 2015b). Like other stigmas, its effects are felt in terms of discriminatory exclusions or mistreatments across many aspects of daily life. Thus, it restricts access to quality health care (Phelan et al. 2015; Puhl and Heuer 2010), creates a significant wage gap in the United States (Colls and Evans 2014; Puhl and Heuer 2009, 2010), and constricts friendships and other types of social support (Brewis et al. 2011; Schaefer and Simpkins 2014). Moreover, individual efforts to cope with and/or avoid weight‐related stigma often lead to less healthy behaviors associated with elevated chronic disease risk (Puhl and Suh 2015; Vartanian 2008).

Experiencing weight‐related stigma can also be extremely psychologically stressful and hence damaging to mental health in and of itself (Major et al. 2012; Sikorski et al. 2015). Recent research suggests that fat stigma is a major (but not well‐recognized) population‐level driver of obesity and chronic disease (Hatzenbuehler et al. 2013). Indeed, its effect on early mortality may be greater than the effects of extremely high levels of body fat (Sutin et al. 2015).

For most traits that become stigmatized in a certain time or place, the people affected—the stigma‐bearers—have several viable options for coping. They can decide to not disclose the trait to others. Alternatively, they can find in‐group support and protection with “sympathetic stigma sharers” or “wise others” (Goffman 1963). Masking morbid obesity is impossible, however, unless one disengages from public life entirely or dispenses with in‐the‐flesh encounters. Even if such masking were feasible, survey studies suggest that people with high body weight are as likely to endorse stigmatizing beliefs about obesity as everyone else and, as a result, they develop less in‐group social support and emotional protection (e.g., Schwartz et al. 2006).

This study addresses the question of how cultural norms around the moral undesirability and unacceptability of fatness exert such a seemingly powerful effect on embodied health. Others have written about “fat embodiment,” although to date this term has been insufficiently theorized and explored (we also agree with Lupton [2013] that some of the writing emerging from fat studies and Health at Every Size movement activists actually separates you from your body in ways that reify Cartesian dualism). Several exceptional pieces of writing on this subject have, however, not only provided detailed (often autobiographical) narratives engaging with experienced, interpersonal stigma (e.g., Braziel and Lebesco 2001; McCullough 2013; Owen 2015; Rothblum and Solovay 2009), but have also engaged deeply with theory in the process.

We have found McCullough (2013) especially helpful in this regard, as she deploys Goffman's (1963:4) use of “abominations of the body” and “blemishes of individual character” to shape a discussion of her own experiences navigating a medical system while “fat and knocked up.” She highlights the ways in which her systematically poor treatment at the hands of a myriad of medical staff resulted from their perception of her body as not only disgusting and risky (an abomination) but also as indicative of a flawed immoral personality (blemished character)—a conflation, as she points out, that reads the body as indicative of the self. In her narrative, she asks if an embodied self is simply “being in the world” (Bourdieu 1992; Csordas 1993, 1994, 2002), what happens to a self that has been identified as fat in a society that engages in profound, systematic discrimination of fat bodies?

In this article, we aim to extend the discussion of experienced fat stigma beyond a focus on the interpersonal. We propose that part of the reason for this stigma's pernicious inescapability lies in part in people's chronic exposure to stigmatizing environmental cues in public spaces. Certainly, fat stigma is constantly reinforced by everyday interactions with others (Greenhalgh and Carney 2014; Hatzenbuehler et al. 2013; Martin et al. 2008; McCullough 2013; Pescosolido 2013). At the same time, however, evidence indicates that spatial and structural factors are also important. One small ethnographic study based in New York City (Meleo‐Erwin 2015), for example, used previously existing research on disability and “misfitting” within the built environment (e.g., Garland‐Thomson 2011) as a springboard to examine the ways in which “fat embodiment” is shaped by failures to physically fit within public spaces. Similarly, the results of a telephone survey with 141 Australians identified as having “high body weight” (Lewis et al. 2011) indicated that spatial and structural forms of stigma hurt people more in the long run than overt interpersonal stigma precisely because the sources of the former are so insidious. Other research has noted that too‐small clothing and too‐tight seating worsens people's anxiety, dissatisfaction, and sense of not belonging (Christiansen et al. 2012; Colls 2006).

A body's navigation of public spaces thus may act to create or reinforce feelings of fat stigma, but any detailed analyses on this specific point have yet to be conducted, although references to such experiences abound in more informal, autobiographical accounts. There is extensive parallel research within disability studies on “disabling environments” or “disabling architectures” (Chouinard et al. 2010; Colls and Evans 2014; Cooper 1997; Link and Phelan 2001; Livingston 2000), and this is relevant to general theory building. Such research, along with the policies that have developed from it, identifies the misfit that individual bodies experience in a specific place that “does not sustain the shape or function of the body that enters it,” ensuring such bodies are “cast out” (Garland‐Thomson 2011:594). The notion of a stigmatizing misfit between specific bodies and specific types of built environments has not yet carried over into thinking about misfitting as a form of fat stigma.

To begin to understand the impacts of what we call “fat stigmatizing environmental cues,” we have focused on the narratives provided by people who have personally experienced such cues: 35 participants who have lived with what is technically known as “morbid obesity” (see Brewis [2011] for a discussion of the BMI classification system; see McCullough and Hardin [2013], Trainer et al. [2015b], and Yates‐Doerr [2012, 2015] for a discussion of the acknowledged problems with the BMI classification system). Our study also includes a broader survey of 296 respondents drawn from the same clinical population. With these perspectives in mind, we consider how felt fat stigma results from the constant difficulties faced in navigating almost every aspect of modern urban space in the United States. In the process, this article expands medical anthropology approaches to fat by honing in on interactive stigmatizing environmental cues, offering ethnographically informed specifics about the processes by which stigma operates through conversation and the built environment. Throughout, we foreground the voices of people who have actually experienced such cues on a daily basis.

Analytically, we distinguish between three types of fat stigmatizing environmental cues that people may be exposed to as they navigate public spaces. Physical–spatial cues refer to difficulty in physically fitting in the normal‐sized world (e.g., seats and seat belts that do not fit). Public attitudinal display cues devalue fat, for example, via negative sentiments expressed on bumper stickers or posted in open online forums. Public reaction cues occur in public spaces and include being ignored, stared at, or treated rudely by strangers in restaurants, supermarkets, or when walking on the street. The latter are also interpersonal and provide a means to examine the ways in which physical–spatial cues are amplified by interpersonal stigma in public spaces. Importantly, both cues and the conversations about those cues may differ, based on such variables as age and gender.

Our overarching aim here is not new within medical anthropology: We wish to unpack the ways in which the physical environment—in particular, the discrimination quite literally built into the physical environment—registers on individual bodies. Our focus on the complex processes by which fat stigma within the environment becomes embodied in the fat bodies misfitting in U.S. public spaces, however, adds an important layer to preexisting work on fatness and obesity, even among those works that have discussed fat embodiment (e.g., Braziel and Lebesco 2001; McCullough 2013; Owen 2015; Rothblum and Solovay 2009). At a time when contentious discussions about public health, the “obesity epidemic,” and the pathologization of large bodies are occurring across the social sciences (e.g., Bell et al. 2011; Braziel and Lebesco 2001; Campos 2004; Greenhalgh and Carney 2014; Hardin 2015; Kulick and Meneley 2005; LeBlasco 2011; Lester 2007; Lupton 2013; McCullough and Hardin 2013; McNaughton 2013; Mendenhall 2012; Moffat 2010; Rothblum and Solovay 2009; Unnithan‐Kumar and Tremayne 2011; Yates‐Doerr 2012, 2015) and beyond, we contend that it is vital to critique the specific choices within planning, policy, and industry operating in the United States that create exclusionary and stressful spaces for a significant number of Americans who attempt to use and live in them.

## Study Population and Methods

Our study population was drawn from the patients enrolled in the main bariatric surgical practice of a national integrated health system. To qualify for initial entry to the hospital program, patients are required to have a body mass index (BMI) minimum of 35, but the range of patient BMIs extends upward into the 80s.

### Ethnographic Population, Methods, and Analysis

The ethnographic phases of the study, including detailed interviews and extensive participant observation, were conducted with the current patient population at just one of the bariatric clinic sites (Arizona). Patients were recruited into the study prior to bariatric surgery or in the 24 months post‐surgery. Like the survey population, therefore, patients in the qualitative analysis (*N* = 35) were either still considered obese (BMI > 30) or normal weight (BMI of 18–24.9), but all had a recent history of morbid obesity. The interview sample was fairly reflective of the overall bariatric clinic population in terms of gender (75% female), ethnicity (25% identified as non‐white, non‐Hispanic), and age (ranging from late 20s to early 70s).

Interviews were all conducted by Sarah Trainer and supplemented with participant observation by Alexandra Brewis and Trainer in multiple contexts within the clinic, in public spaces, and with clinic staff and administrators over a four‐year period. Participant observation with patients included attending pre‐operative classes (held once a week for eight‐week cycles and required for all patients) as well as in the monthly post‐surgery bariatric support group meetings. The interview protocol covered a range of domains around weight and related stigma. Interviews took 45–120 minutes and were audiotaped and then fully transcribed using standard protocols (McLellan et al. 2003).

Transcripts were coded and analyzed using a thematic analysis following the approach of MacQueen et al. (1998). In this analysis, we focused on codes for “structural stigma” (which encompassed physical–spatial cues and public attitudinal display cues) and “interpersonal stigma” (which included public reaction cues) (Krippendorff 2012). The codebook included detailed definitions, typical exemplars, atypical exemplars, and marginal/irrelevant examples from the texts to illustrate the range of meanings assigned to themes. We assessed interrater reliability of codes using a random sample of 40 segments from our preliminary interviews, and final code definitions reached a high level of interrater agreement (kappa >0.7). Core analytic tools included thematic comparison (Bernard and Ryan 2010; Boeije 2002).

### Survey Population, Methods, and Analysis

All patients who underwent bariatric surgery within the health care system in the previous 60 months were invited to participate in the survey phase. The survey sample thus included people still considered technically obese (BMI > 30) and people clinically classified as being at a healthy weight (BMI of 18–24.9) with a recent clinical history of morbid obesity. The final sample size of those who participated in the survey was 296 (40% of the total clinical population). About 77% of respondents were female, and the majority were non‐Hispanic Whites (93%). The average age at the time of bariatric surgery was 52. The demographics roughly match that of the total clinical population in terms of age and gender, although minorities were underrepresented among survey respondents.

Specific items in the survey addressing stigmatizing environmental cues are shown in Table 1 (see below, under Results) and were drawn from the Stigmatizing Situations Inventory (SSI), a validated scale (Puhl and Brownell 2006). For the statistical analysis, responses for the items in each domain were collected on a 4‐point Likert‐type scale (categorized as: never, once, a few times, often). This provided a possible value range between 0 and 9 for the physical–spatial cues; between 0 and 6 for the public attitudinal display cues; and between 0 and 28 for public reaction cues. The overall level of stigmatizing experience is the sum of the values from the three cues. To compare the level of stigmatizing experience by gender, age groups, and body size, we used analysis of variance. *Proc GLM* with ss3 option in SAS handled the unbalanced data structure.

**Table 1:** Descriptive Statistics of the Experience of Stigmatizing Environmental Cues (1)

	In the last 3 months			Any time prior to surgery
	BMI <30		BMI> = 30	(all BMI >30)
*Physical–Spatial Cues*				
Not being able to find medical equipment in a size that works for you	9/289 (03.1%)	8/133 (06.0%)		95/290 (32.8%)
Not being able to fit comfortably into seats on airplanes or in public places	30/289 (10.4%)	25/133 (18.8%)		226/293 (77.1%)
Not being able to find clothes that fit	62/289 (21.5%)	40/133 (30.1%)		247/294 (84.0%)
*Public Attitudinal Display Cues*				
Seeing bumper stickers, t‐shirts, advertising, and so on that ridicule fat people	113/288 (39.2%)	57/132 (43.2%)		167/289 (57.8%)
Felt stigmatized or discriminated against online/on social media by strangers	18/276 (06.5%)	12/26 (09.6%)		72/271 (26.6%)
*Public Reaction Cues*				
Felt stigmatized or discriminated against by servers at restaurants because of your weight	30/288 (10.4%)	22/131 (16.8%)		140/283 (49.5%)
Felt stigmatized or discriminated against by strangers in public places because of your weight	51/287 (17.8%)	37/130 (28.5%)		180/284 (63.4%)
Being offered fashion advice by strangers	40/292 (13.7%)	23/133 (17.3%)		75/291 (25.8%)
Being sexually harassed (cat calls, wolf whistles, etc.) because of your weight	26/292 (08.9%)	12/133 (09.0%)		62/291 (21.3%)
Being stared at in public because of your size	44/292 (15.1%)	29/133 (21.8%)		168/290 (58.0%)
When eating in public, being told “You really shouldn't be eating that because of your size”	8/288 (02.8%)	6/132 (04.6%)		82/289 (28.4%)
Having strangers suggest diets to you	24/290 (08.3%)	20/134 (14.9%)		124/292 (42.5%)
In the supermarket, having people criticize or make comments about your food choices	17/291 (05.8%)	11/134 (08.2%)		76/294 (25.9%)
Overhearing other people making rude remarks about you in public	28/291 (09.6%)	20/134 (14.9%)		150/290 (51.7%)
When walking outside, having people drive by and laugh or shout insults	10/290 (03.5%)	8/133 (06.0%)		108/286 (37.8%)

## Results

### Ethnographic Findings

**Figure 1:**
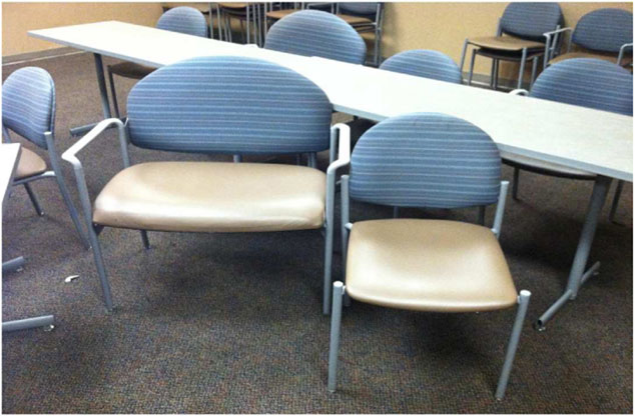
An extra‐wide chair with armrests sits to the left of a regular‐size chair in a behavioral change class at the hospital.

The theme of failure to fit into the physical space one inhabits every day because of one's size emerged in many different contexts: in individual interviews, in discussions during the required behavioral change classes pre‐surgery, and in the support group meetings. Participants detailed a litany of problematic and embarrassing encounters with spindly chairs, narrow restaurant spaces, unwanted gym mirrors, and narrow seating. They also discussed the complex planning required to avoid getting into and out of low‐slung vehicles, maneuvering around crowded working spaces, or walking great distances. Many such instances were noted even during interviews within the clinic. The endocrinology unit provided extra‐wide seats for the behavioral change classes and for patients who visit the endocrinology space specifically, but elsewhere in the hospital and clinic spaces patients do not have such options (see Figure 1).

The university spaces, where some follow‐up interviews were conducted, made no accommodations for size: Participants faced tight parking spaces, a long, hot walk between the parking garage and campus, and private work and public spaces equipped only with standard‐issue chairs. Stopping for coffee before or after an interview involved navigating tight spaces full of undergraduate students, standing in long lines, and sitting at tiny closely spaced tables. Participants never complained about these difficulties, but they often made observations about them.

Within the interviews, themes of interpersonal stigma were reported by most of the participants. Participants also reported many instances in which they experienced stigmatizing environmental cues. Both men and women experienced such cues in apparently roughly equal proportions. Participants with BMIs between 35 and 40 typically reported fewer daily such reminders of misfitting than did those with much higher BMIs.

Mack, a 55 year‐old man who identified as white, made a comment typical of the participant reports of the difficulties involved in negotiating physical space—and the other people inhabiting it—while big.

I was tired of being 450 pounds. It's a lot of weight to carry around. People who aren't heavy don't know the amount of work that's involved in actually being heavy. They think you're fat and lazy. Well, you're maybe lazy from the standpoint of you don't want to do exercise, but it's a lot of work to carry around that much weight. It's our daily lives is a lot of work. People take for granted just bounding up the stairs. When you're 450 pounds, you just don't go bound up the stairs, you know? Or you wonder why you take the elevator versus taking the stairs. Well, because it's a lot of work to walk up those stairs, you know? … I tell people the story about being heavy and going out for dinner. My wife and I would go out for dinner, right? And for the last five years, okay, when you walk into a restaurant. … But when you go into a restaurant, table for two, I need a table. I never wanted a booth because I may or may not fit in a booth depending on how the booth is. And so I didn't want them putting me in a booth because what if I didn't fit in the booth and the table doesn't move? Some booths, the tables don't move.

Restaurant spaces were difficult for many participants: On the one hand, chairs often were too small; on the other hand, booths were often too difficult to get in and out of.

Andrew, 30, was over 25 years younger than Mack and identified as Latino whereas Mack identified as white. But Andrew made observations that showed strikingly similar experiences to those of Mack in this regard. He said:

Clothes didn't fit … had to buy a new belt, had to buy a new shirt, whatever the case may be. … I want to be able to walk into any store and say, “Give me that one right there,” and my size is there. And it isn't a, “Well, let's see if we have it,” or the jacket's too long. That subconsciously is a big thing for me of being able to shop anywhere. I haven't been able to do that consistently for a long time, so it is one of those things that's driving me …

The last time I flew, I was closer to 390, 400, so I was maybe a quarter‐inch from getting the extender piece. And in my head that day, I'm like, “Just no extender piece, just not the extender.” And I barely did it. But I was so close to that that. … It scared me because I can't afford two plane tickets in that sense.

The fact that Andrew was well over 6" tall exacerbated many of the difficulties he faced in this regard. That said, airplane seats were perhaps the most discussed issue of misfitting in our ethnographic data set, in both interviews and group settings.

Alice, also 30 and over 6" tall, but female and white, talked extensively about the misfit between her body and the health care facilities where she worked as an OR nurse:

Mentally, I'm kind of broken. In the OR, you have to not touch sterile equipment and get through—like those rooms really aren't that big. And you know, you have to crawl under the table to get pedals, random stuff. It's just affecting. … It's not like you're just sitting there on a computer. You're standing and bending and moving. And yeah, I can totally tell I'm too fat for this job. I say that all the time. Which is not necessarily true, but … I guess I make plenty of comments like, “Oh, I'm too fat for the OR. Oh, I'm too fat for this job.” Like I say that plenty of times. And it's more of a, “Yeah, I know I'm fat. You don't need to point it out.”

Alice makes the additional point here that not only does she feel that she doesn't fit into her work space, but also that she needs to constantly confess this fact out loud to her coworkers, to mitigate what they may or may not be thinking.

Patricia's narratives explore the notion of misfitting, and the underlying negotiations that this necessitates, in great detail. Because of her age (65+) and impaired mobility as a result of severe joint pain and fibromyalgia, she was already at a disadvantage navigating physical space; her weight (she was severely obese by her own and the program's standards) made the navigation exponentially more difficult. This is clear from the following interview excerpt:

[My husband] and I have always been social people, but it was always having them come to our home because we just like to do it. We would go to other people's homes. … And going into their home, I was always careful enough never to presume where to sit, so it wasn't always weight related. It became more weight related when I was harder for me to get around and harder for me to find a chair that would fit my behind. … I also know if I broke something, I'd feel bad. And I wouldn't want to put them in a position of having to—I remember something and I can't remember what it was I broke, it was something I stepped on. And it was one of the little toys. It was a wooden, handmade toy by his grandfather. I felt so sad. … And all I could do was apologize. … It became harder for me to put myself and them into that kind of a scenario, so we've done more just entertaining here so that nobody had to go through it.

What also emerges in this narrative is that Patricia developed a range of coping mechanisms for managing such situations, often simply by apologizing, as she does above, or by self‐curtailing her social life and ventures outside. Among participants, apologies and self‐curtailment were commonly reported responses to misfitting.

Less common, however, were some of Patricia's other coping mechanisms. She managed her physical space, for example, to a very advanced degree. This was true when she did go to friends’ houses, according to her own report:

And if it's something where I don't have to embarrass or put someone I know ill at ease so they don't have to continually deal with it, but I won't do that to one of my friends. And I'll tell them, “Well, do you have something that's firm enough that I feel comfortable sitting in?” rather than putting it on them. And they'll say, “Yeah, I think I've got something.” And then we can both be comfortable.

She also thought ahead about space negotiations when she went out in public:

Again, because I control the environment I put myself in … you go into a restaurant and you have to squeeze between two tables with the chairs. I would opt for the one that was not in that kind of venue. Ron wouldn't always think about it, but I'd always tell him, “I'm going to take that chair.” “Why?” “It'll be easier.” “Oh, okay.” And just coping skills. I think everybody needs to know what they're dealing with. I have a friend with no arm, you know, and there's just coping skills you learn. Sad to say, obesity is one of those. It's nice to know that there's an option out.”

In this regard, Patricia is very strategic in navigating physical–spatial and public attitudinal cues that her body does not belong. When it was impossible for her to truly manage the public space around her, for instance while traveling, Patricia fell back on humor:

[My husband] loves to travel and so do I. And I've been the anchor, so I'll just have to weigh anchor and go. And I hate getting into airline seats and having to ask for the extender belt. … I tell two funny stories. On the cruise we were on … they had to use one of those smaller boats that you get onto when you're on the big boat. So I was stepping down into that smaller boat and the water rises and falls and you have to time it. And I thought I had it timed, but I didn't. And so there were two men that were helping me. I can still see the look on their faces when they realized I was going to fall and they were going to have to catch me. I even heard one of them kind of groan a little bit because I'm a heavy woman. And no, I didn't get hurt and I thanked them a lot and I said, “I am so sorry.” I said, “If I weren't so big, that wouldn't have been such a problem.” And they said, “Oh, don't worry about it, ma'am.” But I knew it was. I said, “I'll probably be one of your funny stories in the future.” And I'll tell you what, it's okay with me. It was funny. So I had to—and they were from the ship. I thought they were from the dock. So we took another catamaran excursion a day later and my husband was with me this time. And I get ready to go on the boat to transport us where the catamaran is docked and I saw these guys and I started laughing. And he couldn't tell, my husband thought I was laughing and they were laughing, and my husband's totally out of the loop. And I said, “Are you ready?” And they were laughing and saying yes.

Here, she registers many of the same difficulties other participants do—discomfort, lack of mobility, problems with the airplane seat, etc.—but she preempts critique by not only apologizing to others but also by laughing at herself. This tactic was much less common among participants, many of whom did not see the humor in chronically misfitting in public, in front of audiences.

After weight loss, many participants continued to be sensitive to previously difficult public spaces, including ones that they now navigated with more ease. Mack, for example, reported that eight months after surgery (and after losing 145 pounds), he could now sit at a booth, but it was not something he took for granted:

Post‐surgery, my wife and I went out to dinner at Chili's and they put us at a booth. And I sat down at the booth no problem. And my wife is—this is funny now. She was so happy, she got her phone out, she took a picture of me. “What are you doing taking a picture of me.” “It's a big deal. You're in a booth.”

### Survey Findings

Reporting retrospectively regarding exposure to stigmatizing cues prior to surgery (Table 1), survey respondents reported noticing cues in all categories. Frequency ranged from 21.3% who experienced weight‐related sexual harassment to 84% being unable to find clothes that fit. Notably, the two highest reported exposures to stigmatizing cues are both physical–spatial: clothes and seating. Tests for differences in reporting (summarized in Table 2) show no significant variation by age or gender. Level of obesity, however, clearly predicted sensitivity to cues. People with increasing levels of obesity prior to surgery reported higher level of exposure to stigmatizing cues in all three domains.

**Table 2:** Comparing the Level of Stigmatizing Environmental Cues by Gender, Age Group, and BMI at Any Time before Surgery

	Physical–Spatial Cues	Public Attitudinal Display Cues	Public Reaction Cues	Overall
*By Gender*				
Male	4.81	1.93	8.95	18.92
Female (ref.)	4.46	1.60	7.34	16.60
Total number of cases	281	263	259	239
*By Age Group at Surgery*				
Between 20 and 30 years old	4.95	2.52**	10.67**	21.61**
In the 40s	5.50**	2.34**	11.98**	23.96**
In the 50s	4.96*	1.92**	9.34*	19.56**
Over 60 years old (ref.)	4.04	1.21	5.38	12.75
Total number of cases	279	261	257	237
*By BMI at Time of Surgery*				
Less than 40	3.26**	1.50*	6.58**	13.90**
Between 40 and 45	4.59**	1.86	8.01**	17.36**
Between 45 and 50	4.80**	1.60*	7.84**	16.86**
Over 50 (ref.)	6.37	2.23	11.34	24.30
Total number of cases	281	258	258	236

We observed significant differences in the recent reporting (where the time frame was the “last three months”) of exposures to physical–spatial cues based on current weight status. Among those currently obese (BMI over 30), a high percentage reported experiences of stigmatizing physical–spatial cues within the previous three months. In addition, when we compared those who currently had BMIs greater than 35 with those with lower BMIs, reports of exposure to these types of cues lessened as BMI reduced through weight loss (Table 3). Again, there was no difference by age group or gender.

**Table 3:** Comparing the Level of Stigmatizing Environmental Cues by Gender, Age Group, and BMI in the Last Three Months

	Physical–	Public Attitudinal	Public Reaction	
	Spatial Cues	Display Cues	Cues	Overall
*By Gender*				
Male	0.58	0.75	1.34	3.21
Female (ref.)	0.68	0.95	1.58	3.78
Total number of cases	277	267	270	245
*By Current Age Group*				
Between 20 and 30	0.64	1.19*	1.98*	4.28*
In the 40s	0.65	1.17*	2.37**	4.88**
In the 50s	0.73	0.87	2.09**	4.37**
Over 60 (ref.)	0.62	0.65	0.47	1.99
Total number of cases	275	265	268	243
*By Current BMI*				
Less than 27.5	0.31**	0.77†	0.81**	2.35**
Between 27.5 and 35	0.49**	0.85	1.21**	3.20**
Over 35 (ref.)	1.53	1.11	3.47	6.98
Total number of cases	279	265	270	244

*Note*: *p < .05; **p < .01, two tailed.

Reports of negative public reactions by strangers in public spaces significantly was reduced in survey respondents with BMIs below 35. There was no gendered difference, but older people were less likely to report noticing cues. In contrast, public attitudinal display cues (e.g., fat shaming in advertising) showed no statistically significant reduction in sensitivity to noticing cues among those with BMIs below the obese and even the mid‐overweight thresholds (30 and 27.5 BMI, respectively). There was no difference in this sensitivity to public attitudinal display cues by gender, but younger age groups reported encountering these more often. Importantly, noticing advertising and similar such cues remained frequently reported even in respondents who had lost enough weight to no longer be considered technically obese (39.4%).

## Discussion

Both the interview and the survey findings show that physical space is important in terms of producing feelings of discomfort and shame over body and size. Airplane seats were a hot‐button issue in the ethnographic data, and confirmed in the surveys, with many participants struggling with the challenge at least once in their lives. Most strikingly expressed in interviews, perhaps, is the degree of worry, shame, and blame that people feel about “not fitting,” the degree of planning and organization that goes into avoiding situations of not fitting, and the expressed need to be “the jolly fatty” (in the words of Lesley 2011) when not fitting is unavoidable.

Our findings resonate with prior, small‐scale ethnographic studies. The participants in Meleo‐Erwin's study (2015) all identified New York City as particularly stigmatizing due to densely populated small spaces and crowded public transportation. The theme of having to pre‐plan trips outside the home and anxiously anticipate any potential areas of difficulty in fitting runs through our own narratives—with participants talking about how they must constantly “scan” (to use Kirkland's [2008] term). That this theme ran through so many narratives even though urban Phoenix has far more open space than New York City is striking. So, too, is the fact that such experiences of exclusion from the built environment occurred across gender and age cohorts in our qualitative and quantitative sampling. Notably, the frequency and type of misfitting did show some differences that were sensitive to gender and age in the survey data. Younger women seemed especially sensitive to the clothing cues. This also parallels scholarly arguments that fat bodies are also raced, classed, and gendered, and that these other identity markers profoundly influence experiences of weight‐related stigma (Bordo 1993; Garland‐Thomson 2011; Fikken and Rothblum 2012; Meleo‐Erwin 2015).

In *The End of Normal*, Davis (2014) argues that bodies are inherently biocultural—i.e., they are complex embodiments of technological, sociocultural, historical, and biological processes and that current notions of normal bodies may be more accepting of body diversity but still systematically exclude bodies perceived to be pathological. While Davis's focus is on disabled bodies, this same essential point holds true for bodies designated pathologically, morbidly obese. Much of the focus in medical anthropologists’ writing about obesity and fatness thus far has provided important ethnographic perspectives on the ways in which biomedicine and public health have pathologized obesity (e.g., Hardin 2015; McCullough 2013; McCullough and Hardin 2013; Yates‐Doerr 2012, 2015), but in this article we demonstrate that space, architecture, clothing, and so on can also render bodies pathological through systematic exclusion. The men and women we spoke with reported a constant bombardment of cues that their bodies—and therefore their embodied selves—did not fit appropriately in daily urban life as they knew it. McCullough (2013:228) writes: “Fat embodiment becomes that much heavier as it bears the weight of the medical and social gaze as a spoiled body and a spoiled person,” but in this instance, the gazes combine with environmental cues in a particularly toxic fashion.

At the crux of the definition of stigma is the notion of a moral discrediting (Goffman 1963; Pescosolido 2013). Because obesity is viewed as an individually produced, disgusting state by many Americans, there is little sympathy expressed for people struggling to fit into too‐small spaces and places (Farrell 2011; Kirkland 2008; Rogge 2004). Indeed, one of the most disturbing aspects of the misfitting reported in the interviews is the impatience and lack of sympathy people often reported receiving from others witnessing their struggles as well as an underlying notion that the struggles to fit in were somehow deserved punishment for being too large and/or would goad people into losing weight (Longhurst 2005). This observation provides a different means to look at the quantitative findings—suggesting the co‐occurrence of physical–spatial with interpersonal cues may be especially important to what people *feel* is most stigmatizing. Thus, physical–spatial cues are very important in creating exclusion, but the effects of these are exacerbated when combined with an apparent lack of sympathy or understanding from the audience watching the physical struggle. Moreover, as some of the conversations with participants reflect (particularly the quote from Alice), people also engage in fat talk (Nichter 2000; Taylor 2015) about their misfitting that self‐stigmatizes, adding another layer of stress and self‐blame to their experiences.

## Conclusion

Our own research confirms universal awareness of and sensitivity to a range of fat‐stigmatizing cues that exist in public spaces, and these add substantively to a person's felt stigma when living with a very high body weight. Interviewees and survey respondents reported they constantly managed their navigation of such environments. While our current research focuses on individuals whose label of “morbid obesity” propels them into bariatric surgical programs, their daily battles with misfitting into public space, both physically and socially, resonate widely (e.g., Lesley 2011; West 2013) and need to be understood as a major factor in the underlying processes by which high weight becomes embodied as poor health. In this particular instance, chronic experiences of battling too‐small spaces, clothes, vehicles, furniture, and so forth not only resulted in heightened reported distress but also made people less likely to leave the relative comfort of their own homes. Of course, this decreased activity then leaves them even more vulnerable to charges of laziness—a moral failing already widely attributed to people perceived as fat within American society generally (e.g., McCullough 2013). A practical finding of this study, therefore, is the need to highlight and address the powerful and negative effects of anti‐fat messaging, not just in traditional media and advertising but also in the built environment itself, and to articulate to a wide audience that empathetic public reactions to people's weight‐related struggles greatly matter.
